# Arthritis in a Glyptodont (Mammalia, Xenarthra, Cingulata)

**DOI:** 10.1371/journal.pone.0088646

**Published:** 2014-02-13

**Authors:** Fernando Henrique de Souza Barbosa, Kleberson de Oliveira Porpino, Ana Bernadete Lima Fragoso, Edison Vicente Oliveira

**Affiliations:** 1 Departamento de Geologia, Universidade Federal de Pernambuco, Recife, Brazil; 2 Departamento de Ciências Biológicas, Universidade do Estado do Rio Grande do Norte, Mossoró, Brazil; Friedrich-Schiller-University Jena, Germany

## Abstract

Arthritic lesions have been frequently diagnosed in the fossil record, with spondyloarthropathy (a type of erosive and pan-mammalian arthritis) being one of the most common types described to date for mammals, though not restricted to this group. Here, we identify spondyloarthropathy in fossil bones from the late Pleistocene in Brazil assignable to a large glyptodont individual. Bone erosions in the peripheral joints (viz., the ulna, radius, left femur and tibiae-fibulae) associated with osteosclerosis allow the diagnosis of spondyloarthropathy. The presence of osteophytes in seven bones of the forelimbs (viz., the ulna and radius) and hind limbs (viz., the tibiae-fibulae, left femur and patellae) and a subchondral cyst in one element (viz., the left femur) indicate secondary osteoarthritis. A calcified deposition on the articular surface of the left patella indicates the presence of calcium pyrophosphate deposition disease, which, like the observed osteoarthritic alterations, likely represents a complication of spondyloarthropathy. This is the first report of spondyloarthropathy for xenarthrans.

## Introduction

Arthritis includes a wide variety of joint diseases, including those in which the proliferation of new bone is the main feature (osteoarthritis) and those in which the key characteristic is bone erosion [1], [2]. Among the latter type, spondyloarthropathy is one of the most common pathologies. It is an inflammatory form of arthritis encompassing several diseases that share common morphological and immunological alterations [1], [2]. It is characterized by the propensity to form new bone, to ossify insertion sites of tendons or ligaments, to erode or asymmetrically fuse peripheral joints and to present articular lesions in the vertebral column and the sacroiliac joint [1]–[3]. Such alterations are associated with a specific histocompatibility antigen, HLA-B27 [1]–[3]. It has been recognized in several groups of fossils and living mammals, ranging from marsupials to proboscideans, perissodactylans, and primates, among several others [1], [4], from the Paleocene to the present [5]. However, its earliest appearances in the fossil records are in archosaurs from the Triassic and Jurassic periods [6], [7].

In the Pleistocene in Brazil, arthritics lesions in xenarthrans, a mammalian group formed by sloths, anteaters, armadillos, giant armadillos and glyptodonts, have been sparsely documented [8]–[10]. In this paper, we report a case of spondyloarthropathy in a glyptodont, one of the most abundant and diversified members of the Pleistocene megafauna of South America. This account represents the first documentation of spondyloarthropathy in large xenarthrans. In addition, this finding broadens the geographic distribution of spondyloarthropathy in extinct mammals, insofar as the previous well-documented reports of this disease have been restricted to North America, Europe and Asia [5].

## Materials and Methods

### Geological Background

**Figure 1:**
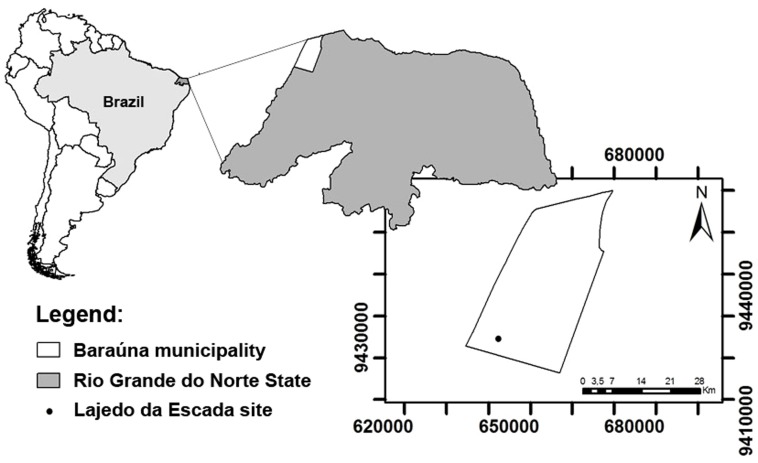
A location map of Lajedo da Escada site in Baraúna municipality, Rio Grande do Norte state, Brazil.

The glyptodontid remains studied here were collected together with bones of other mammalian taxa in a limestone cave in the Lajedo da Escada site, Rio Grande do Norte state, Brazil (Fig. 1), during expeditions in the late 1960s. The Lajedo da Escada site (UTM 642516, 9424502) is composed of two karstic pavements formed by carbonate rocks belonging to the Jandaíra Formation (Apodi Group, Potiguar Basin) with a total area of 5 km^2^ [11]. In these pavements, there are several small caves filled with clayish sediment. The fossil accumulations in these sediments are composed mostly of mammalian remains belonging to species typical of the Quaternary of the Brazilian Intertropical Region [12]. Comparable findings known from other continental fossil accumulations in deposits in northeastern Brazil (viz., in caves and tank deposits [13]) have traditionally been dated as Late Pleistocene [12]. This inferred age concurs with most absolute age estimations of bones collected in caves and tank deposit accumulations from this region [14], [15].

### Specimens Studied and Taxonomic Assignment

The material studied is housed in the Onofre Lopes Vertebrate Paleontology Collection of the Museu Câmara Cascudo, Natal, Rio Grande do Norte state, Brazil, and was released for this study by the curator in charge of this collection (M.F.C.F. Santos). It includes twenty-seven postcranial bones that were found in association with elements of an exoskeleton during the original excavation [11]. The elements of the exoskeleton are composed of osteoderms from the dorsal carapace and caudal rings assigned to the Glyptodontinae genus *Glyptotherium* [16]. The morphology of the postcranial bones is compatible with that of Pleistocene Glyptodontinae genera and contrasts with the morphology of representatives of other genera found in the Pleistocene deposits of northeastern Brazil, such as *Panochthus* and *Hoplophorus*, by presenting features such as comparatively short metapodials, a calcaneus with the peroneal tubercule being absent and a short, wide sulcus for the calcaneal tendon of the gastrocnemious muscle [17], [18]. Because there are no other remains of glyptodonts at the Lajedo da Escada site and because all glyptodontid material was found in association with the aforementioned osteoderms, it appears more parsimonious to assign the postcranial bones studied here to the same genus as the osteoderms. Apart from the fact that these postcranial elements were collected in association, we believe that they belong to a single individual for two additional reasons: a) among them, there are several paired postcranial homologous bones presenting comparable dimensions (viz., the right and left patellae, the left and right tibiae-fibulae, the right and left calcanei, the right and left naviculars and the right and left radii; see below) and several articulating bones forming part of a hind limb (viz., the left patella, left tibia-fibula and left femur) and a forelimb (viz., the right radius and right ulna); and b) an examination of the epiphysis–diaphysis fusion grade (to the long bones) and the centrum–disk fusion grade (to the vertebrae) suggest that all analyzed bones are in the same ontogenetic stage (viz., a mature stage).

The postcranial elements studied include the following: MCC 176-177-178-182-410-415-V metapodials; MCC 232-237-238-239-240-242-V caudal vertebrae; MCC 337-V fragment of a pelvis; MCC 389-V left navicular; MCC 464-V right patella; MCC 467-V fragment of a right navicular; MCC 473-V left patella; MCC 481-V right radius; MCC 482-V proximal half of a left radius; MCC 485-V right calcaneus; MCC 491-V right ulna; MCC 862-V axis; MCC 1087-V proximal half of a right humerus; MCC 1515-V left tibia-fibula; MCC 1555-V left calcaneus; MCC 1560-V left femur; and MCC 1561-V right tibia-fibula.

### Paleopathological Analysis

All specimens were analyzed through a visual inspection. Additionally, the specimens MCC 473-V (left patella), MCC 481-V (right radius), MCC 482-V (proximal half of a left radius), MCC 491-V (right ulna) and MCC 1560-V (left femur) were submitted to a radiologic evaluation using an X-ray apparatus (Siemens Heliophos 4 S, 500 Ma, 125 Kv). The greater trochanter and a small portion of the diaphysis of the left femur MCC 1560-V, as well as the entire fibular portions of the left (MCC 1515-V) and right (MCC 1561-V) tibiae-fibulae, were restored in plaster. These restored parts were not taken into account in our analysis.

## Results

Among the examined skeletal elements, eight presented pathological lesions: the right patella (MCC 464-V), the left patella (MCC 473-V), the right radius (MCC 481-V), the proximal half of the left radius (MCC 482-V), the right ulna (MCC 491-V), the left tibia-fibula (MCC 1515-V), the left femur (MCC 1560-V), and the right tibia-fibula (MCC 1561-V).

### Forelimb elements

**Figure 2:**
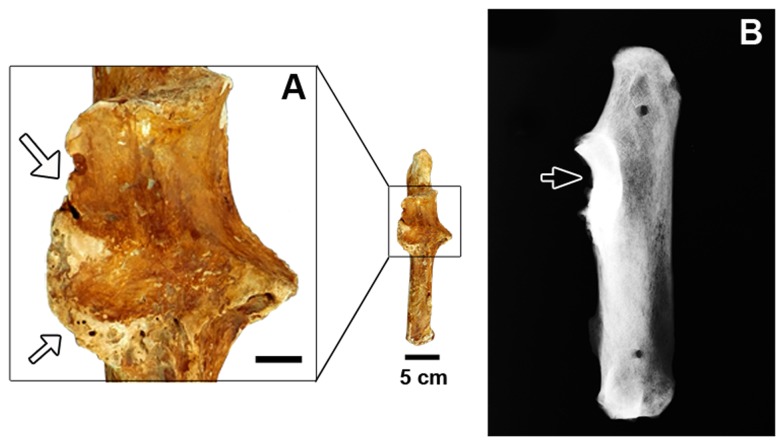
Right ulna of *Glyptotherium* (MCC 491-V). (A) A detail of the trochlear and radial notch in a cranial view; (B) Lateral radiography. The large white arrow indicate bone erosion, the smaller white arrow indicates bone growth and the black arrow indicates sclerosis. Scale bar in the detail A = 1 cm.

**Figure 3:**
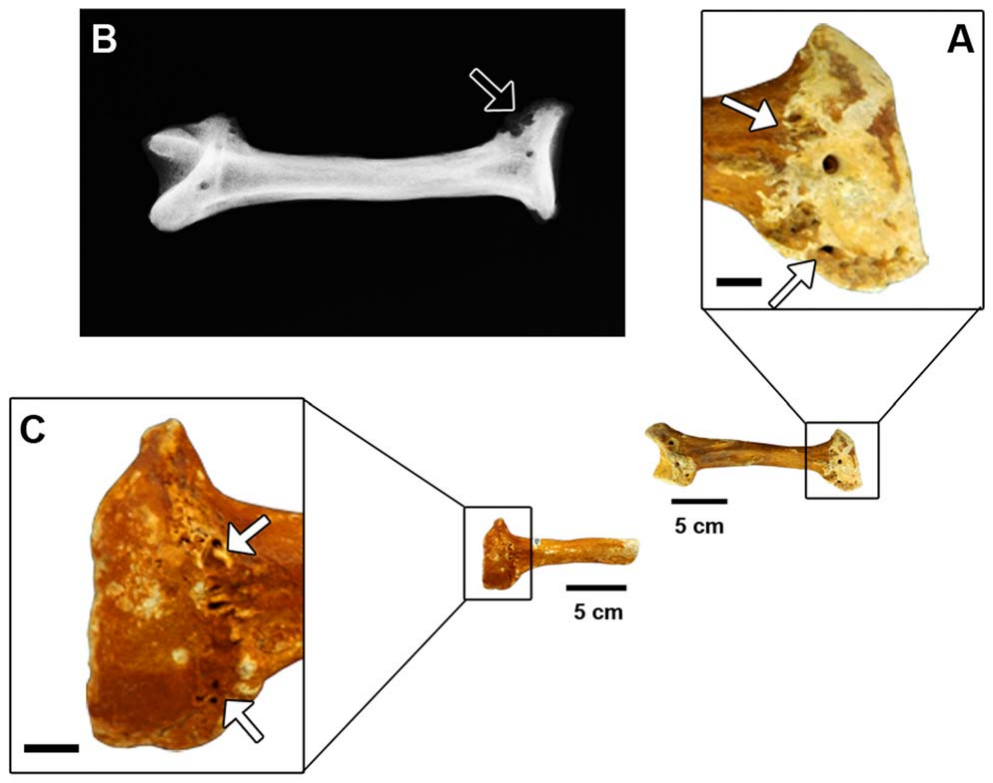
Radii of *Glyptotherium*. (A) A detail of the proximal epiphysis of the right radius MCC 481-V in caudal view; (B) Cranio-caudal radiography of the right radius MCC 481-V; (C) A detail of the proximal epiphysis left radius MCC 482-V in caudal view. Scale bars in the details A and C = 1 cm.

The right ulna shows bone erosion and remodeling on the lateral surface of the proximal epiphysis extending from the most proximal portion of the trochlear notch to the distal margin of the radial notch (Fig. 2A). Its medial border also shows some bone growth. The lateral border of the articular surface for the radial notch of the ulna in the right radius shows remarkable bone erosion and remodeling, which extends medially (Fig. 3A). The eroded region shows smooth, rounded edges, with small, shallow, elliptical holes. In the left radius, similar alterations are observed in the homologous articulation, but they are less remarkable than in the right radius (Fig. 3C). In the X-ray images, it is possible to recognize sclerosis in the affected articular surfaces of both the right radius and ulna, represented by areas with increased bone density marked by enhanced radiopacity (Fig. 2B and 3B).

### Hind limb elements

**Figure 4:**
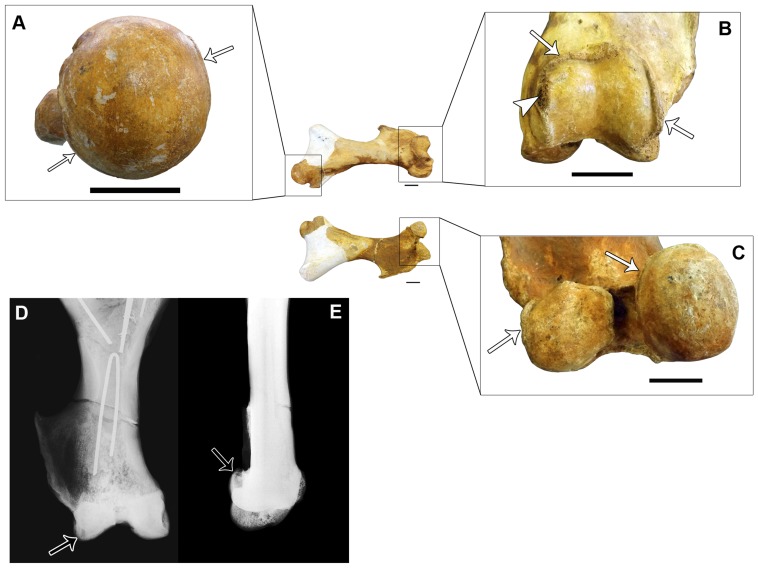
Left femur of *Glyptotherium* (MCC 1560-V). (A) A detail of the femoral head in proximal view; (B) a detail of the trochlea in cranial view; (C) A detail of the condyles in caudal view; (D) Cranio-caudal radiography of the femur; (E) Lateral radiography. The white arrows indicate osteophytes, the white arrowhead indicates bone erosion and the black arrows indicate subchondral cysts. Scale bars  = 5 cm.

Cranially, the left femur presents bone growth in almost all margins of the head and of the trochlea (Fig. 4A and 4B). Additionally, there is well-marked bone erosion in the medial border of the trochlea and small pits on the proximal and lateral portions of its surface (Fig. 4B). Caudally, bone growth similar to that on the head is observed on the margins of the lateral and medial condyles (Fig. 4C), with some differences concerning their extension: in the former, the growth is limited to the lateral and proximal borders; in the latter, it is observed along all borders except for a small area on the distal border. The radiographic evaluation of the left femur reveals a small lytic lesion in the lateral condyle, which likely represents a degenerative subchondral cyst (Figs. 4D and 4E).

**Figure 5:**
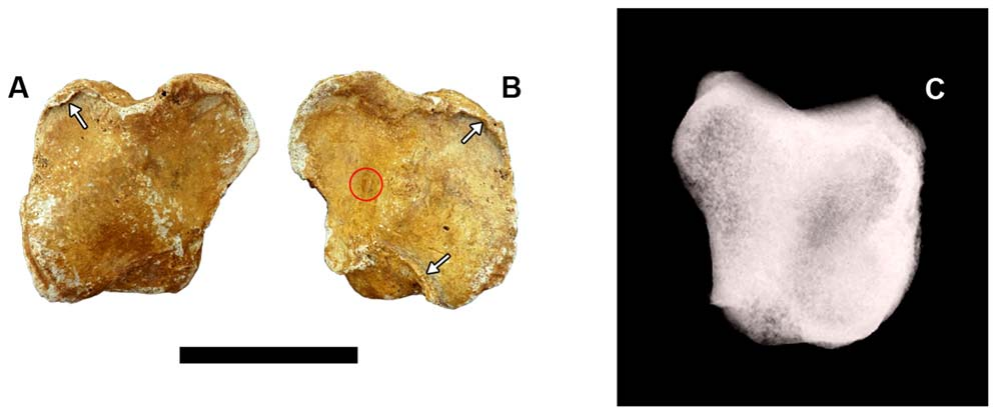
Patellae of *Glyptotherium*. (A) Right patella MCC 464-V in caudal view; (B) left patella MCC 473-V in caudal view; (C) Cranio-caudal radiography of the left patella. The white arrows indicate osteophytes, and the red circle indicates calcium deposition. Scale bar  =  5 cm.

In the patellae (Figs. 5A-C), bone growth is present along the margins of the articular surface of the femoral trochlea. It is more evident in the right patella than in the left one because, in the latter, the borders of the articular surface were strongly eroded by taphonomic processes (Fig. 5B). In the X-ray images, scleroses are less noticeable (Fig. 5C) in contrast to the macroscopic evidence. Additionally, in the left patella (MCC 473-V), there is a small calcific deposition on the articular surface of the trochlea next to the lateral border (Fig. 5C).

**Figure 6:**
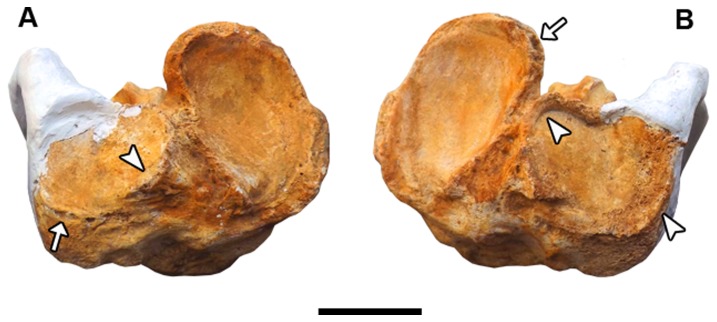
Tibiae-fibulae of *Glyptotherium*. (A) Right tibia-fibula MCC 1561-V in proximal view; (B) Left tibia-fibula MCC 1515-V in proximal view. The arrows indicate bone erosion, and arrowheads indicate osteophytes. Scale bar  =  5 cm.

In the tibiae-fibulae, erosions and new bone formations are present on the preserved borders of the lateral and medial condyles (Fig. 6A and 6B). On the other hand, the astragalar facets of both tibiae-fibulae present no sign of bone alteration.

## Discussion

Despite the fact that spondyloarthropathy is a type of arthritis characterized mostly by erosive lesions in the vertebral column and the sacroiliac joint, it can be identified by the presence of asymmetrical pauciarticular peripheral joint erosion or fusion in cases where there are no vertebral or sacroiliitis lesions [2], [19]. We have identified well-marked erosive lesions characteristic of spondyloarthropathy on the trochlear notch of the right ulna, the lateral border of the articular surface of the right radius for the radial notch of the ulna and on the homologous articulation of the left radius, as well as on the borders of the lateral and medial condyles of the tibiae-fibulae. On the right ulna and on the right and left radii, these degenerative alterations are surrounded by irregular bone growth. Additionally, the X-ray images allowed the recognition of bone sclerosis associated with these lesions on the articular surfaces of the forelimb, evidencing the occurrence of bone reaction in those elements.

As previously mentioned, spondyloarthropathy is a type of arthritis that encompass several varieties of arthritis: ankylosing spondylitis, Reiter’s syndrome, psoriatic arthritis, arthritis associated with inflammatory bowel disease and undifferentiated spondyloarthropathy [1], [2]. However, to differentiate among the types of spondyloarthropathy is not always possible, especially when the evidence is based only on skeletal remains. When the available material is limited to bones, as in most paleontological cases, the distinction is based on the observation of patterns of bone alterations in the joints of the spine and the sacroiliac joint [1], [2]. Unfortunately, the only preserved components of the axial skeleton associated with the forelimb and hind limb elements described here are the caudal vertebrae, which present no noticeable alterations.

When spondyloarthropathy is not associated with vertebral and sacroiliac lesions, it can be confused with rheumatoid arthritis (a polyarticular, erosive arthritis) [2], [19], [20]. However, bone-reactive sclerosis at the borders of the lesions, as evidenced by the X-ray images of the affected skeletal elements studied here (Figs. 2B and 3B), is absent in rheumatoid arthritis [2]. Moreover, subchondral erosion, like that observed in the left femur, is rare in rheumatoid arthritis [2], thus allowing us to eliminate it as the cause of the observed lesions.

Interestingly, all of the specimens analyzed present osteophytes on at least one of their articular margins—the sole exceptions were the ulna and radii, where the observed bone reactions may not be osteophytes but, instead, the reactive bone typical of spondyloarthropathy (Fig. 2 and 3). The presence of osteophytes in synovial joints is characteristic of osteoarthritis, a non-inflammatory and non-erosive type of arthritis [2]. Moreover, there is a subchondral cyst in the lateral condyle of the left femur (Fig. 4D and 4E), a type of lesion that has been related to complications or an advanced stage of osteoarthritis [21]. The absence of grooved and eburnated articular surfaces suggests that the osteoarthritis was not severe, and it may have developed as a secondary complication to the spondyloarthropathy, which can be clearly differentiated from osteoarthritis by the presence of erosive lesions; therefore, spondyloarthropathy can be considered the primary disease in the studied case. In addition, it is worth noting that osteoarthritis is quite uncommon in fossil and free-ranging living mammals and is apparently more characteristic of artificially restrained animals [2], [19].

The presence of calcific concretions on the joint surface of the left patella (Fig. 5B) is compatible with calcium pyrophosphate deposition disease (CPPD). This disease is a type of crystalline arthritis recognized by the presence of a calcified sheet deposited on the articular surface, radiocarpal articular surface indentation and calcific concretions on the joint surface [2], [22]. In the present case, CPPD, like the osteoarthritis, seems to be a secondary phenomenon—i.e., such alterations represent a complication of spondyloarthropathy.

Spondyloarthropathy has been associated to individual (e.g. body mass) and ecological (e.g. life history traits) characteristics of mammalian species [23]. Previous studies on several extinct and extant mammalian groups [19], [24] suggest that spondyloarthropathy is a pan-mammalian disease but not directly related to body mass. However, a recent comparative study revealed statistically significant results for spondyloarthropathy as more prevalent in large-bodied primates and carnivores [23]. The overweight caused by the large body mass is an appealing explanation for the presence of spondyloarthropathy in the large-bodied glyptodont studied here. In fact, large-bodied mammals are also more exposed to infections [25] for several reasons, including greater food consumption (thus increasing the risk of ingesting pathogenic organisms) and stress related to joint damage that could facilitate the development of local infectious disease [23]. Nevertheless, further studies including cingulates with different body masses would be necessary to test the correlation of spondyloarthropathy/large body mass in this group.

Concerning previous arthritis cases in large Xenarthrans and other representatives of the South American megafauna, there are some previous identifications, such as osteoarthritis in the vertebrae of Megatheriidae [8], in a right humerus of *Mylodon* [9] and metacarpals of *Panochthus* [10] as well as osteochondritis dissecans in a left radius and left femoral head of *Toxodon*, a right femur of *Mylodon* and a pelvis fragment of *Glyptodon* [9]. In some of these reports [8], [9], subchondral erosions on facets joints are described as one of the main lesions identified. This type of lesion is more compatible with spondyloarthropathy than with osteoarthritis (which is a non-erosive disease) [2] or osteochondritis dissecans. Although spondyloarthropathy may be the likely diagnosis for some of the aforementioned reports of arthritic lesions, a more detailed analysis of the referenced material would be crucial for a sound conclusion. Thus, the spondyloarthropathy case reported in this paper is, to our knowledge, the first formal description of this disease for large Xenarthrans and for the South American megafauna.

## Conclusions

Visual examination and radiologic analysis of several postcranial bones assignable to a single individual of *Glyptotherium* from Lajedo da Escada site, northeastern Brazil, evidenced three types of arthritis: one type of inflammatory arthritis (spondyloarthropathy), one type of crystalline arthritis (calcium pyrophosphate deposition disease) and one proliferative arthritis (osteoarthritis). Spondyloarthropathy, considered the primary disease, is diagnosed by the presence of bone erosions in a right ulna, right and left radii, a left femur and both right and left tibiae-fibulae and is associated with bone sclerosis (in the right ulna and radii). Calcium pyrophosphate deposition disease was diagnosed by the presence of calcium deposition observed on the articular surface of the left patella. Finally, osteoarthritis was identified by the presence of osteophytes in the left femur, in the right and left patellae, and in both tibiae-fibulae. This represents the first case of spondyloarthropathy formally reported in Xenarthrans.
